# CuPt Alloy Thin Films for Application in Spin Thermoelectrics

**DOI:** 10.1038/s41598-019-40021-x

**Published:** 2019-02-28

**Authors:** Kun Tian, Ashutosh Tiwari

**Affiliations:** Nanostructured Materials Research Laboratory, Department of Materials Science and Engineering, University of Utah, Salt Lake City, Utah 84112 USA

## Abstract

Spin thermoelectrics represents a new paradigm of thermoelectricity that has a potential to overcome the fundamental limitation posed by the Wiedmann-Franz law on the efficiency of conventional thermoelectric devices. A typical spin thermoelectric device consists of a bilayer of a magnetic insulator and a high spin-orbit coupling (SOC) metal coated over a non-magnetic substrate. Pt is the most commonly used metal in spin thermoelectric devices due to its strong SOC. In this paper, we found that an alloy of Cu and Pt can perform much better than Pt in spin thermoelectric devices. A series of CuPt alloy films with different Pt concentrations were deposited on yttrium iron garnet (YIG) films coated gadolinium gallium garnet (GGG) substrate. Through spin Seebeck measurements, it was found that the Cu_0.4_Pt_0.6_/YIG/GGG device shows almost 3 times higher spin Seebeck voltage compared to Pt/YIG/GGG under identical conditions. The improved performance was attributed to the higher resistivity as well as enhanced spin hall angle of the CuPt layer.

## Introduction

Realization of smart thermoelectric generators (TEG) that can efficiently generate electricity from waste heat has been a dream for the material scientists since long^1^. Heat to electricity conversion efficiency of conventional thermoelectric devices is limited by the energy losses resulting from the heat conduction and joule dissipation in the thermoelectric materials. Efficiency of a thermoelectric system is quantified by a dimensionless quantity known as the figure of merit, ZT = TS^2^σ/κ^1^. Where ‘T’ is the absolute temperature, ‘S’ is the Seebeck coefficient, ‘σ’ is the electrical conductivity, ‘κ’ is the thermal conductivity of the material. Any improvement in the efficiency requires a reduction in the thermal conductivity (κ) and an increase in the electrical conductivity (σ) of the thermoelectric material. However, these two requirements are contradictory as the thermal conductivity of thermoelectric materials is dominated by the electronic component of thermal conductivity (κ_e_), and according to the Wiedmann-Franz law, *κ*_*e*_ ∝ *σ*^1–5^. This limits the possibility of any significant improvement in the efficiency of conventional thermoelectric systems. Over the last several decades, efforts have been made to overcome the above limitation by using nanotechnological approaches; however, only modest improvements could be achieved^6,7^. It is widely believed that these small incremental advances to the current TEG technology will not be sufficient for developing next generation high-efficiency thermoelectric devices. Such development will require the utilization of entirely new and transformative approaches. Spin thermoelectric represents one such approach^2–4,8–12^.

Most of the important research in spin thermoelectrics started after the pioneering work of Kirihara and Uchida *et al*. in which they showed that a temperature gradient can induce spin voltage in a layer of magnetic insulators via a phenomenon known as spin Seebeck effect (SSE), which in turn can be converted to an electrical voltage by an attached metallic layer of a high spin-orbit coupling metal via ISHE mechanism^5,13–20^. In the above scenario, the heat flow occurs in the insulating layer while the charge current transport in the metallic layer, so the electrical and thermal transport properties of the system can be optimized independently. Based on this concept they demonstrated a thermoelectric coating comprising of bismuth doped yttrium iron garnet (Y_2_Fe_5_O_12_, YIG) and Pt. These films were coated on a (111)-oriented gadolinium gallium garnet (Gd_3_Ga_5_O_12_, GGG) substrate. It was found that when a temperature difference ΔT is applied between the top of the Pt film and the bottom of the GGG substrate, it drives a spin current density, ***j***_***s***_, at the Pt/YIG interface perpendicular to the plane. The above process is followed by the conversion of ***j***_***s***_ into an electric field E_ISHE_ by the inverse spin-Hall effect (ISHE) in the Pt following equation^10,14^:

1 $${{\boldsymbol{E}}}_{{\boldsymbol{ISHE}}}=({\theta }_{SH}\rho ){{\boldsymbol{j}}}_{{\boldsymbol{s}}}\times \frac{{\boldsymbol{M}}}{|{\boldsymbol{M}}|}$$

where *θ*_*SH*_ and *ρ* are the spin Hall angle (SHA) and electrical resistivity of the non-magnetic metal (NM), and ***M*** is the magnetization of YIG layer. If ‘*l*’ is the length of the metallic layer, the above field will result in a spin Seebeck voltage given by the expression:

2 $${V}_{SSE}=|{{\boldsymbol{E}}}_{{\boldsymbol{ISHE}}}|l$$

As can be seen from equation (1) and (2), the strength of the spin Seebeck voltage is directly proportional to the SHA of the NM used in the device.

Pt is the most widely used metal in spin Seebeck devices due to its large SHA that arises due to strong intrinsic spin-orbit coupling present in the material^21–23^. However, Pt is a very expensive metal and hence there has been a quest to find alternate materials that are less expensive and at the same time can exhibit similar or higher SHA than Pt. Several recent studies have suggested that large SHA can be realized in metallic alloys due to extrinsic SO interaction resulting from impurity scattering mechanisms, namely skew and side jump scattering mechanisms^24–40^. In this regard, a number of metallic alloy systems have been found to exhibit comparable SHA as pure Pt. For example, Niimi *et al*. obtained a large SHA by introducing Ir impurities in Cu matrix^29^. In another study, they also observed a large SHA in CuBi alloy^30^. Laczkoxski *et al*. reported large SHA in AuW alloy for 7% W^38^. Obstbaum *et al*. found a substantial increase of SHA compared to pure Pt or Au^35^. Inspired by these reports, in this study we have investigated CuPt alloy system as a replacement of Pt in spin thermoelectric devices. A series of CuPt alloy films were deposited on yttrium iron garnet (YIG) films coated on gadolinium gallium garnet (GGG) substrate. SSE measurements were performed on these samples as a function of the composition of the CuPt alloy. It was found that the Cu_0.4_Pt_0.6_/YIG/GGG devices show almost 3 times higher voltages compared to Pt/YIG/GGG under exactly identical conditions. The improved performance was attributed to the higher resistivity as well as enhanced spin hall angle of the CuPt layer.

## Methods

CuPt thin film strips (10 mm long, 1 mm wide, 10 nm thick) were deposited on epitaxial YIG thin film (5 µm thick) grown along (111) crystal orientation on Gadolinium Gallium Garnet (GGG, 0.5 mm thick) substrate (purchased from MTI Corporation). The depositions of CuPt films were performed by co-sputtering of Cu and Pt using Denton 635 system at room temperature with the Argon pressure of 5 mTorr and flow rate of 35 sccm. The concentration of Pt in CuPt alloy was varied by adjusting the deposition rate of Pt. A series of CuPt films with the atomic percentages of Pt: 5%, 7%, 10%, 15%, 20%, 30%, 40%, 50%, 60%, 80% and 100% were prepared. The structure of the films was examined using X-ray diffraction (XRD) and transmittance electron microscopy (TEM) measurements (see Figs S1 and S2 in supplementary information section).

**Figure 1:**
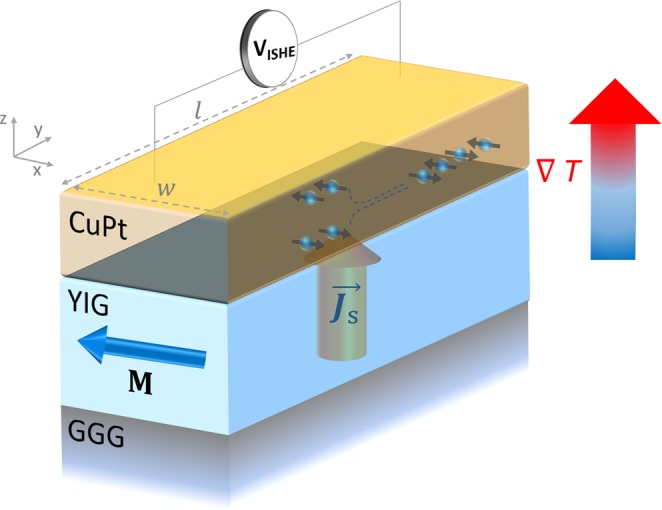
Schematic diagram illustrating the experimental configuration used for measuring longitudinal Spin Seebeck voltage from CuPt/YIG samples.

For spin Seebeck measurements, the CuPt/YIG samples were sandwiched between two AlN plates which provided thermally conducting heat bath. A micro- heater and a Si diode were mounted onto each AlN plate to generate and measure the temperature difference between the CuPt/YIG stack in the z-direction. A magnetic field was applied to magnetize the YIG film in the x-direction, while the resulting ISHE voltage was measured along y-direction by a nano-voltmeter (Keithley 2182 A). The scheme of the experiment configuration is shown in Fig. 1. The electrical resistivity of the CuPt films was measured using the four-probe technique at room temperature.

## Results and Discussion

**Figure 2:**
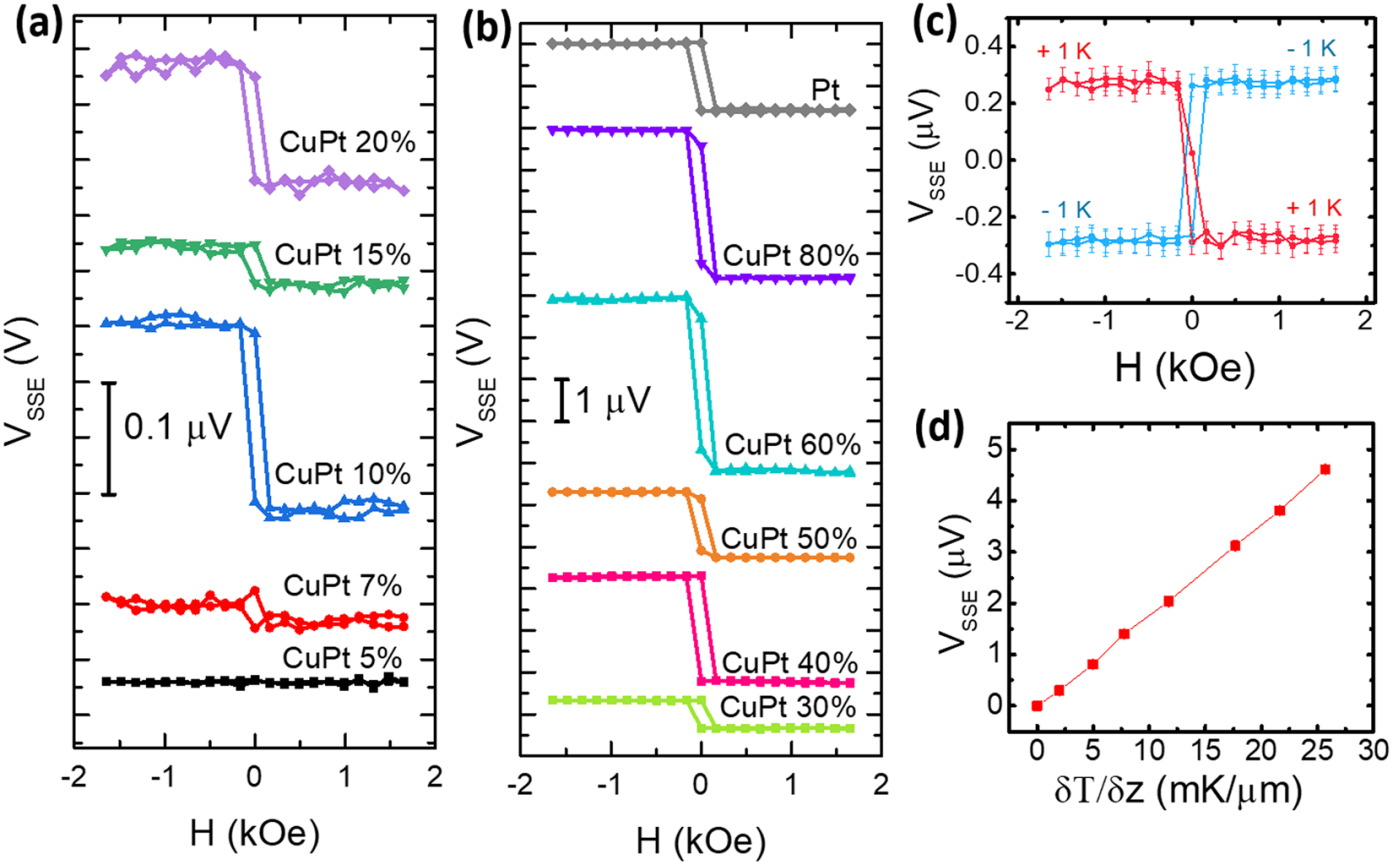
(**a**) SSE voltage measured from CuPt 5% to 20%, and (**b**) SSE voltage measured from CuPt 30% to 100% as a function of magnetic field under a temperature difference of 6 K across CuPt/YIG/GGG interface. (**c**) V_SSE_ as a function of applied magnetic field for ∆T = ±1.0 K at room temperature. (**d**) SSE voltage vs. δT/δz. δT/δz is the temperature gradient across the CuPt/YIG/GGG stack.

In Fig. 2(a,b), we have presented the V_SSE_ vs. magnetic field data recorded from various CuPt/YIG samples. The absolute value of V_SSE_ was determined as the half of the difference of saturation voltages at the positive, V_SSE_(H^+^), and negative, V_SSE_(H^−^), magnetic fields, V = [V_SSE_(H^+^) − V_SSE_(H^−^)]/2. It was found that the sign of V_SSE_ for all the CuPt alloys remains the same as that for Pt^41^. The magnitude of V_SSE_ was found to vary quite sensitively as the composition of the CuPt alloy changed from the Cu-rich regime to Pt-rich regime. A maximum value for V_SSE_ was obtained for the alloy composition of Cu: 40% and Pt: 60%. Figure 2(c) shows the V_SSE_ data for Cu_0.4_Pt_0.6_ sample for ∆T values of +1 K and −1 K. Upon the change of the direction of ∆T, the sign of V_SSE_ is reversed while its magnitude remains almost same. In Fig. 2(d), we have shown the V_SSE_ vs. δT/δz data for the Cu_0.4_Pt_0.6_ sample. δT/δz is the temperature gradient across the whole stack of CuPt/YIG/GGG. A linear dependence can clearly be seen.

**Figure 3:**
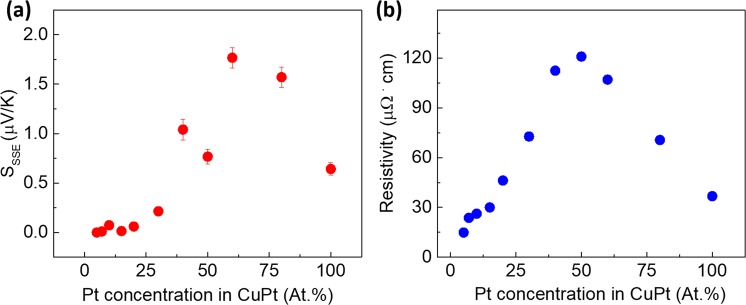
(**a**) The value of normalized SSE coefficient measured from samples of CuPt/YIG with different Pt% ranging from 5% to 100%; (**b**) The resistivity values of CuPt films with Pt % ranging from 5% to 100%.

In literature, Spin Seebeck coefficient (S_SSE_) is defined by E_ISHE_/(δT/δz)^42^. The exact procedure for accurate quantitative determination of S_SSE_ has been elaborated by Uchida *et al*.^43^ and Iguchi *et al*.^44^ In the present study, we calculated the normalized S_SSE_ by using the expression (∆V × t_total_)/(∆T × l), where l and t_total_ are the length of the CuPt strip and the thickness of the substrate, respectively. The resulting values of S_SSE_ for all the samples are shown in Fig. 3(a). As the concentration of Pt in the alloy increases, S_SSE_ first increases and then starts decreasing. The value of S_SSE_ for Cu_0.4_Pt_0.6_ is about 1.8 µV/K, which is almost 3 times the corresponding value (0.6 µV/K) for Pt measured under the identical conditions.

**Figure 4:**
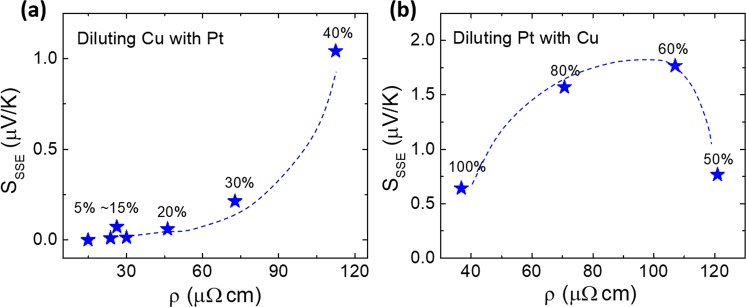
(**a**) S_SSE_ vs. resistivity values of CuPt samples with Pt% from 5% to 40%. (**b**) S_SSE_ vs. resistivity values of CuPt samples with Pt% from 50% to 100%.

Figure 3(b) shows the room temperature electrical resistivity of CuPt films as a function of Pt concentration in the alloy. As can be seen, the resistivity first increases as Pt concentration increases and then drops down. A maximum resistivity value of 121 µΩ·cm was obtained for the sample with Pt concentration of 50%. In Fig. 4(a,b), we have plotted S_SSE_ vs. resistivity values of CuPt samples in Cu-rich and Pt-rich regimes, respectively. As indicated by Equation (1), the value of S_SSE_ should depend directly on the resistivity of the top metal layer. Non-liner dependence of S_SSE_ vs. resistivity curves indicates that in addition to resistivity, the SHA of the metal layer and the spin current density at the CuPt/YIG interface may also be important factors affecting the values of S_SSE_ as the composition of the alloy changes.

In order to understand the experimental data more clearly, we need to look into the complete expression for *S*_*SSE*_. As per the theory of magnon driven spin-Seebeck effect, the value of S_SSE_ is given by the expression^11,45,46^:

3 $${S}_{SSE}={C}_{YIG}{G}_{r}{\theta }_{SH}\rho l\frac{\lambda }{t}{[1+{G}_{r}\rho \lambda coth(\frac{t}{\lambda })]}^{-1}tanh(\frac{t}{2\lambda })$$

where *C*_*YIG*_ is a constant and depends on the properties of YIG^46^, *l* is the distance between voltage contacts, *t* is the thickness of the metal layer, *λ* is the spin-diffusion length of the metal layer, and *G*_*r*_ is the real part of the spin-mixing conductance which determines the efficiency of spin pumping through the YIG/NM interface. A higher spin mixing conductance can enhance the value of S_SSE_ as it determines the spin pumping efficiency. From the reported values of interfacial spin mixing conductance as determined by ferromagnetic resonance (FMR) technique^23^, the value of *G*_*r*_ for YIG/Pt is a few times higher than that for YIG/Cu. Though we do not know the value of *G*_*r*_ for all the compositions studied in this paper, we made an estimate by assuming that value of *G*_*r*_ changes linearly as the composition of the alloy changes from YIG/Cu (1.6 × 10^18^ m^−2^) to YIG/Pt (6.9 × 10^18^ m^−2^)^23^. This is consistent with earlier report by Takizawa *et al*. where they showed that spin mixing conductance in CuIr alloy increases linearly as the atomic % of Ir in the alloy increases^47^. Other important parameter that comes in the expression for S_SSE_ is the spin diffusion length *λ*. For pure Pt, the value of *λ* has been reported to be about 7 nm^22,23^. The value of *λ* for pure Cu is expected to be much larger due to its weaker spin orbit coupling. However, Gradhand *et al*. showed that when Cu is alloyed with metals with strong spin orbit coupling (SOC), the value of *λ* decreases very significantly even for slight doping and becomes almost same as in the metal with strong SOC^28^. Based on the above, it is expected that the spin diffusion length for all the alloy composition studied in this paper will be almost same as for pure Pt.

By using the values of *S*_*SSE*_, *ρ*, *G*_*r*_, and *λ* in Equation (2), the value of SHA can be determined. However, for that we first need to know the value of *C*_*YIG*_. Since the value of *C*_*YIG*_ depends on the YIG, we used our experimentally measured S_SSE_ data for pure Pt and the reported value of *θ*_*SH*_ of 0.1 to estimate the value of *C*_*YIG*_^23^. Once the value of *C*_*YIG*_ is known, the value of *θ*_*SH*_ for all the alloy compositions was estimated using the following expression:

4 $${\theta }_{SH}=\frac{{S}_{SSE}}{{C}_{YIG}{G}_{r}\rho l{[1+{G}_{r}\rho \lambda coth(\frac{t}{\lambda })]}^{-1}\frac{\lambda }{t}\,\tanh (\frac{t}{2\lambda })}$$

**Figure 5:**
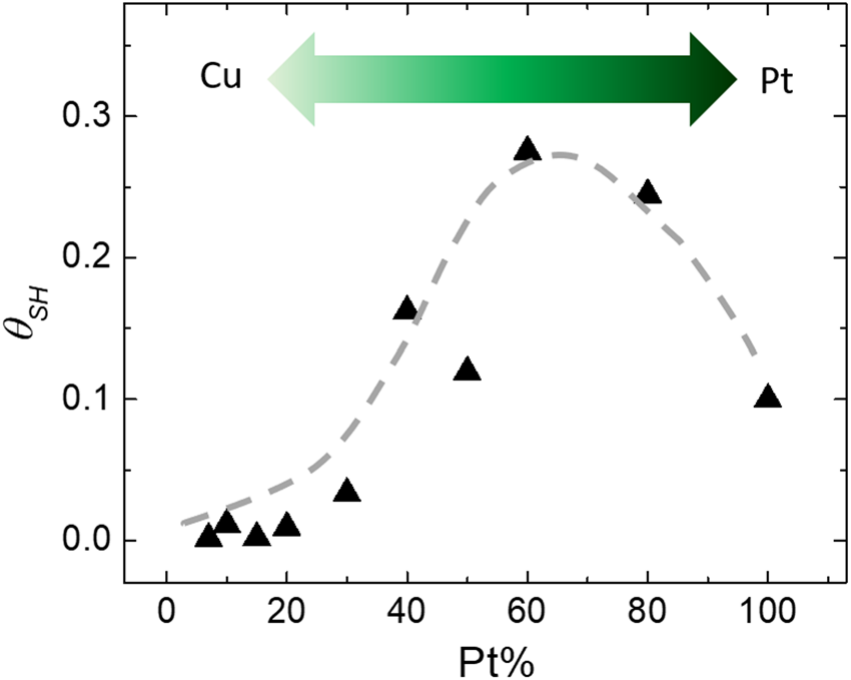
The estimated values of SHA for each sample with different Pt%.

Figure 5 shows the plot of the calculated values of *θ*_*SH*_ as a function of Pt concentration in CuPt alloy samples. Some salient features of this plot are: (1) value of *θ*_*SH*_ for Pt 7% sample was 0.002 which remained more or less constant till the Pt concentration of 20%; (2) for Pt concentration above 20%, a fast increase in the of *θ*_*SH*_ values was observed. Sample with Pt concentration of 60%, showed the highest *θ*_*SH*_ value of 0.27; (3) on further increasing the Pt concentration, the *θ*_*SH*_ showed a decease reaching a value of 0.1 for the Pt 100% sample. Though the initial increase in the value of *θ*_*SH*_ of CuPt when Pt is introduced in the system is easily understandable as the Pt is known to possess much stronger SOC than Cu, an abrupt increase in the value of *θ*_*SH*_ for Pt concentration of about 20% and the fact that the alloy with Pt concentration of 60% shows even higher *θ*_*SH*_ than pure Pt is very intriguing and suggests a very exciting physics hidden in this system. It is noteworthy that Ramaswamy *et al*. found that the *θ*_*SH*_ of Cu_1−x_Pt_x_, which was determined by the method of spin torque magnetic resonance, showed a similar trend as a function of Pt concentrations in the alloy^40^. They concluded that the reason of high *θ*_*SH*_ of CuPt alloy compared to either pure Pt or Cu could be related to the extrinsic contributions of spin Hall effect from both screw scattering and side-jump mechanisms. From application point of view, the large *θ*_*SH*_ exhibited by CuPt alloy offers great potential for its application in next-generation spin thermoelectric power generators.

### Summary

In summary, we have studied the performance of CuPt alloy films as a replacement of Pt in spin thermoelectric devices. A series of CuPt alloy films with varying compositions were deposited on YIG films and SSE measurements were performed. Using the experimentally measured S_SSE_ and *ρ*, the values spin Hall angle were determined. Among all the samples, Cu_0.4_Pt_0.6_ showed the highest S_SSE_ which is about three times higher than S_SSE_ for the pure Pt measured under the identical conditions. Considering the low cost of Cu compared to Pt ($0.01/g for Cu vs. $28.78/g for Pt as per market price in 2018), almost 3 times higher SSE voltage shown by Cu_0.4_Pt_0.6_/YIG compared to Pt/YIG, is very exciting and has the potential to revolutionize the field of spin thermoelectrics.

## Supplementary information

Supplementary Information

## Data Availability

All data generated or analyzed during this study are included in this published article (and its Supplementary Information files).
