# Supplementary material for: CuPt Alloy Thin Films for Application in Spin Thermoelectrics
Source: Sci Rep. 2019 Feb 28;9:3133. doi: 10.1038/s41598-019-40021-x (PMC6395799; doi:10.1038/s41598-019-40021-x)
Supplement: Supplementary file 1 — Supplementary Information [file 41598_2019_40021_MOESM1_ESM.docx]

**Supporting Information**

**CuPt Alloy Thin Films for Application in Spin Thermoelectrics**

Kun Tian and Ashutosh Tiwari*

**1. TEM, EDAX and XRD results from CuPt films**

Cross-sectional TEM images of some of the CuPt/YIG samples were recorded. XRD measurements were performed to find the lattice parameters. Figure S1(a) and (b) show the high resolution TEM images of the CuPt/YIG interface for Pt 10% and 60% samples, respectively. In both the cases, CuPt layer was found to be comprised of nano-crystals. Nanocrystalline lattices with different lattice orientations were clearly observed in the case of Pt 10% samples. Due to overall high atomic density of the film with Pt 60%, we were not able to achieve an equally good contrast as for Pt 10% sample but the lattice planes from the edge of the film were still visible. The d-spacing of these lattices were measured from the inverse fast Fourier transform (IFFT) images. In the case of Pt 10% sample, d-spacings of ~2.2 Å and ~1.9 Å were observed. The observed d-spacing of 2.2 Å could be from (111) plane of CuPt alloy which is smaller than d_(111)_ of pure Pt (~2.3Å) and larger than d_(111)_ of pure Cu (~2.1 Å). The d-spacing of 1.9 Å could correlate to the (200) plane which is also between the d_(200)_ values for Cu (~1.8 Å) and Pt (~2.0 Å). In the case of Pt 60% sample, a d spacing of ~2.3 Å was observed which could correspond to (111) plane. From the observed lattice spacings, the lattice parameter ‘a’ was estimated using equation of $d=\frac{a}{\sqrt{h^{2}+k^{2}+l^{2}}}$, it is found the lattice parameter $a$ of CuPt 10% equals to 3.8 Å. And a is found to be ~4.0 Å for CuPt 60%.

**
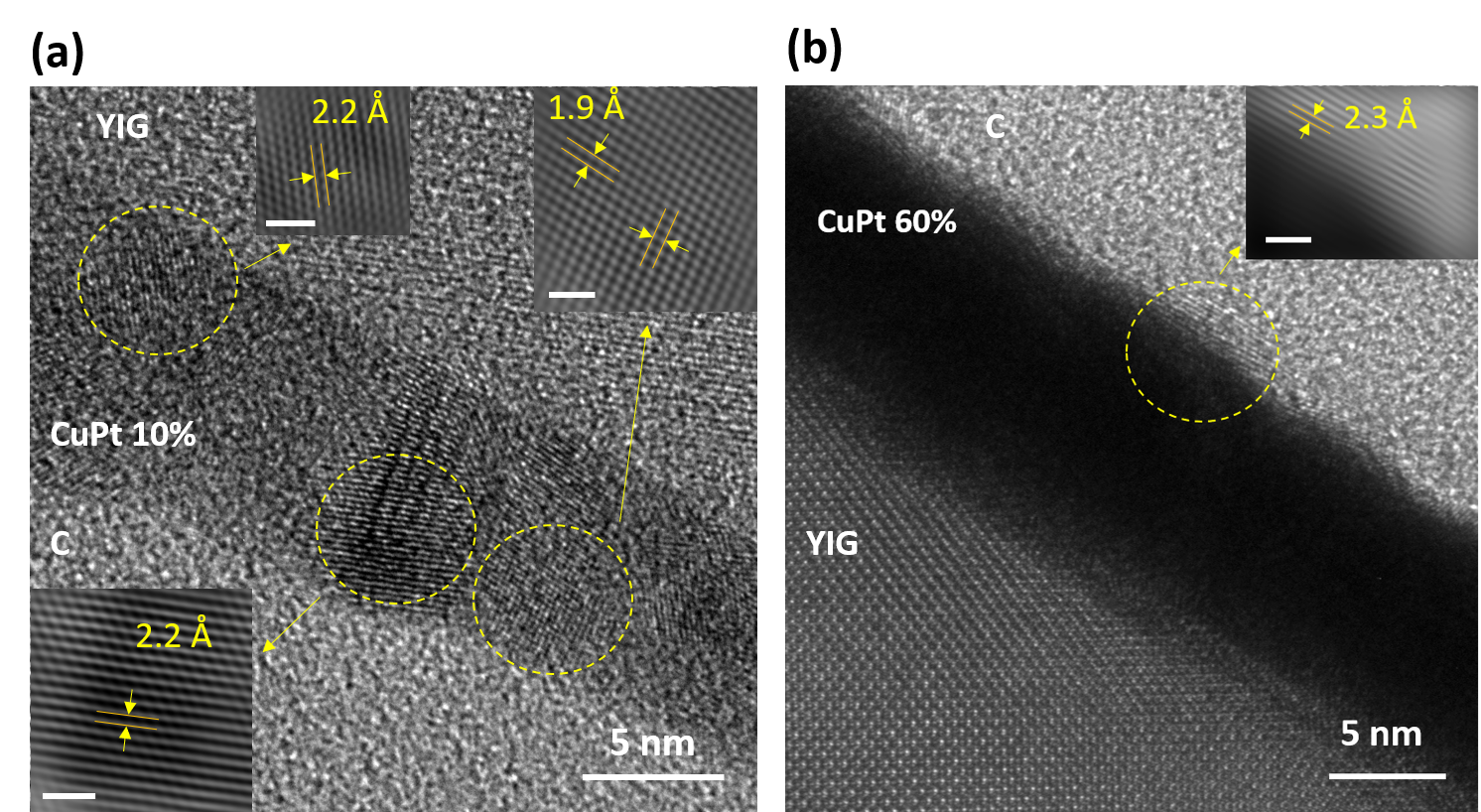
**

**Figure S1** (a) High-resolution TEM images of CuPt/YIG 10%. The enclosed region of yellow-dash circle indicates the short-range crystalline lattices of CuPt alloy. The d-spacing of these lattices were measured through the IFFT images. (b) High-resolution TEM images of CuPt/YIG 60%. The scale bar in IFFT images indicate 1 nm.

Figure S2 shows the XRD data depicting the CuPt (111) peak. From this, the d_111_ spacing in different films were calculated using the Brag’s Law, 2dsinθ=nλ, where θ is the half of the diffraction angle and λ is the wavelength (1.54 Å) of the Cu K-α radiation used in the measurement. From d_111_, lattice parameter ‘a’ for different films was determined. It was found that as Pt concentration increases from 10% to 80%, lattice parameter ‘a’ increases from 3.8 Å to 4.1 Å, which is in good agreement with our TEM results.

**
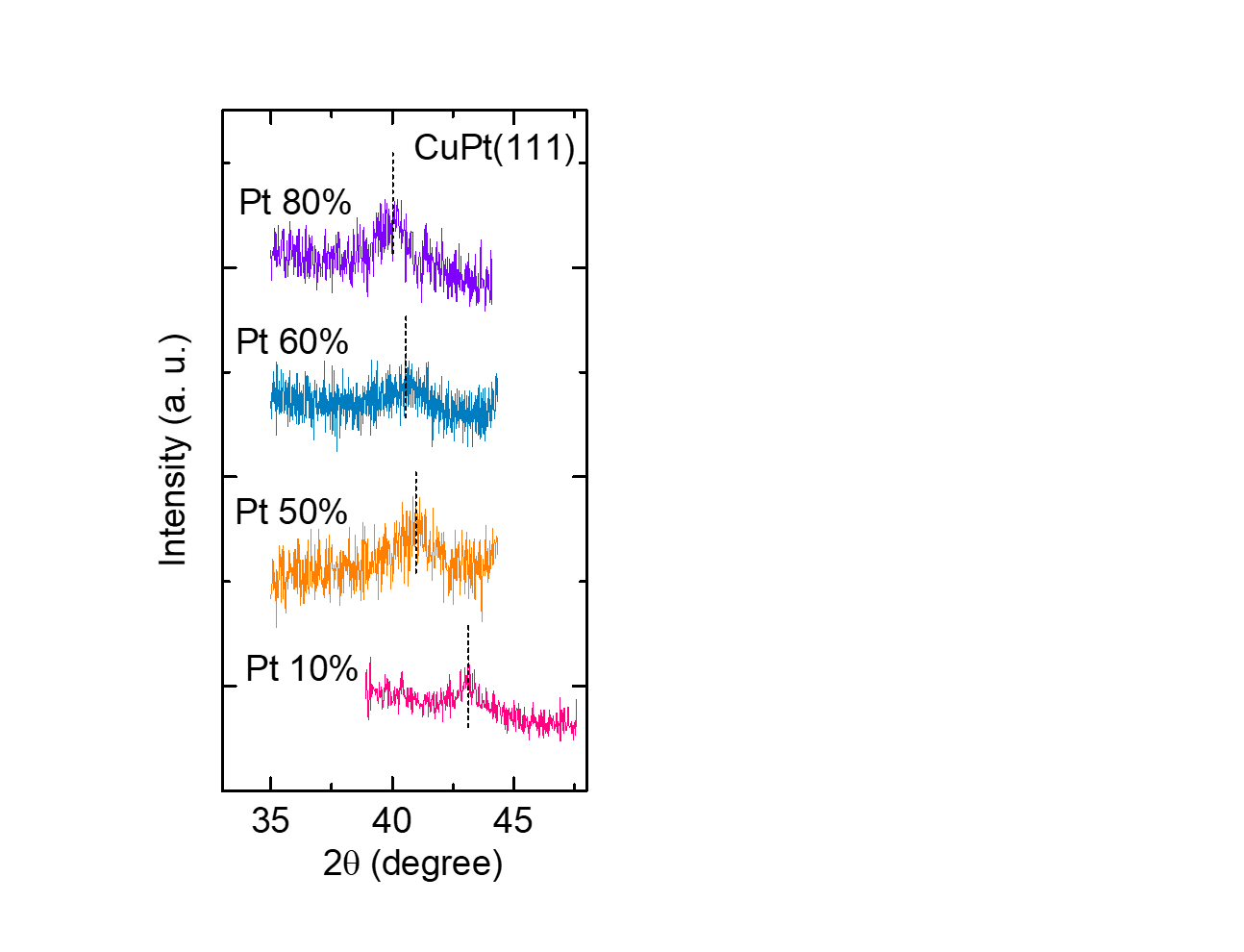
**

**Figure S2** XRD data showing the CuPt (111) peaks. Films with Pt concentration of 50%, 60%, and 80% were 10 nm thick while the film with Pt concentration of 10% was 20 nm thick.
